# Epigenetic control of Epstein–Barr virus transcription – relevance to viral life cycle?

**DOI:** 10.3389/fgene.2013.00161

**Published:** 2013-08-27

**Authors:** Alison J. Sinclair

**Affiliations:** School of Life Sciences, University of SussexBrighton, UK

**Keywords:** Epstein–Barr virus, CpG-DNA methylation, DNA binding, transcription factor, replication cycle, cancer

## Abstract

DNA methylation normally leads to silencing of gene expression but Epstein–Barr virus (EBV) provides an exception to the epigenetic paradigm. DNA methylation is absolutely required for the expression of many viral genes. Although the viral genome is initially un-methylated in newly infected cells, it becomes extensively methylated during the establishment of viral latency. One of the major regulators of EBV gene expression is a viral transcription factor called Zta (BZLF1, ZEBRA, Z) that resembles the cellular AP1 transcription factor. Zta recognizes at least 32 variants of a 7-nucleotide DNA sequence element, the Zta-response element (ZRE), some of which contain a CpG motif. Zta only binds to the latter class of ZREs in their DNA-methylated form, whether they occur in viral or cellular promoters and is functionally relevant for the activity of these promoters. The ability of Zta to interpret the differential DNA methylation of the viral genome is paramount for both the establishment of viral latency and the release from latency to initiate viral replication.

In cellular genomes, the methylation of 5′ cytosines in CpG-dinucleotides leads to recruitment of methyl-DNA binding proteins that co-operate with other epigenetic events to promote the repression of transcriptional activity (reviewed in Wade, 2001; Klose and Bird, 2006; Jones, 2012; Muers, 2013). Although the double-stranded DNA genome of Epstein–Barr virus (EBV) γ herpesvirus resides in the nucleus of human cells and carries the hallmarks of cellular chromatin, the viral genome provides an exception to this rule during the replication phase of its life cycle.

## EPSTEIN–BARR VIRUS ASSOCIATION WITH MAN

Epstein–Barr virus is an almost ubiquitous human virus, which is transferred from person to person in saliva. Infection results in virus entry into both B-lymphocytes and epithelial cells. EBV promotes the proliferation of infected B-lymphocytes and readily generates immortalized cell lines when infection is undertaken in an *in vitro* culture system. The majority of these immortalized cells are recognized by the host immune system and destroyed but some enter the memory B-cell pool, down regulate EBV gene expression and persist in a latent state. Viral latency can be a long-term event and the association of EBV with an infected individual is considered to be for life. EBV is associated with the development of several types of cancer associated with lymphocytes or epithelial cells, principally Burkitt’s lymphoma, Hodgkin’s disease, and nasopharyngeal carcinoma. Primary infection with EBV can also result in infectious mononucleosis (Rickinson and Kieff, 2007).

## EPIGENETIC CHANGES DURING THE EBV LIFE CYCLE

Epstein–Barr virus interacts with cells in a complex manner: the virus is either in a latent state in which only a small sub-set of the viral genes are expressed or it undergoes a lytic replication cycle in which the entire repertoire of EBV genes is expressed and viral progeny are generated (Rickinson and Kieff, 2007). Crucially, the switch from latency to the lytic replication cycle is triggered by physiological stimuli, which can be reproduced in *in vitro* culture systems. It is at this point that the normal epigenetic paradigm is broken.

**FIGURE 1:**
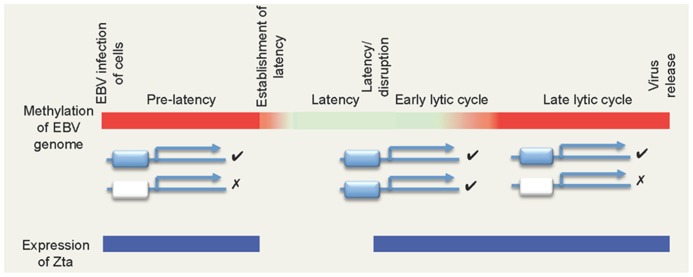
**The biphasic DNA methylation cycle of the EBV genome and its impact of ZREs.** The colored bar depicts the methylation state of EBV genome during different phases of the viral life cycle, with non-methylated DNA in red and methylated DNA represented in green. Two types of Zta-responsive gene are shown below: those containing ZREs that are independent of DNA methylation (blue) and those that are dependent on methylation (white). Note that non-methylated CpG-ZREs cannot be bound by Zta. Periods where Zta is expressed are indicated in blue.

Following infection, the viral double strand DNA genome is established in the nucleus of the cell where it circularizes to form an episome and then replicates once per cell cycle in synchrony with the host genome. During this time, the majority of the viral promoters are silent, with just a few directing the expression of the latency-associated genes. Many studies of individual viral promoters have demonstrated an inverse correlation between promoter activity and the presence of DNA methylation at CpG-dinucleotides within the promoter (reviewed in Minarovits, 2006; Niller et al., 2009). Indeed, recent genome-wide analyses support the contention that the EBV genome is extensively methylated during latency, with only the few active promoter regions spared (Fernandez et al., 2009; Kalla et al., 2010; Woellmer et al., 2012). In contrast, following the onset of the lytic replication cycle, the viral genome becomes largely un-methylated at CpG-dinucleotides (Fernandez et al., 2009). Thus, the majority of the viral genome cycles between an un-methylated and a heavily methylated state (**Figure 1**).

This biphasic methylation state poses an intriguing question. If the promoters of the genes required for lytic replication are silenced by DNA methylation during latency, how is the silencing overturned? There are no reasons to suspect that the mechanisms involved in gene repression are specific to EBV. First, repressive histone modifications, such as the heterochromatin-associated tri-methylation of lysine 9 (H3K9me3) and polycomb-associated tri-methylation of histone 3 at lysine 27 (H3K27me3) marks have been identified on the EBV genome (Murata et al., 2012; Ramasubramanyan et al., 2012b; Woellmer et al., 2012; reviewed in Murata and Tsurumi, 2013). Second, histone remodeling and the appearance of activating marks such as tri-methylation of lysine 4 on histone 3 (H3K4me3) occurs during the latency/lytic cycle transition (Woellmer et al., 2012). Third, and most importantly, sensitive methylation mapping suggests that no change in DNA methylation status occurs prior to the activation of lytic cycle gene expression (Woellmer et al., 2012).

The surprising finding was that the EBV genome requires DNA methylation to reactivate it from latency (Kalla et al., 2010, 2012). This has been fine-mapped to several EBV lytic cycle gene promoters. In comparison with the control of host gene expression, a requirement for DNA methylation at viral promoters presents a paradox. The key to resolving this paradox rests with the unique properties of the EBV-encoded transcription factor, Zta (BZLF1, ZEBRA, Z, EB1).

## THE Zta TRANSCRIPTION FACTOR

Zta is a member of the bZIP family of transcription factors, but it has an unusual dimerization domain, driving the exclusive formation of homodimers (Petosa et al., 2006). Zta contains a classical transactivation domain, which interacts with RNA polymerase II (RNA pol II) associated proteins presumably stabilizing RNA pol II at Zta associated promoters (Lieberman and Berk, 1991). Zta interacts with sequence specific motifs (Zta-response elements, ZREs), resembling AP1 sites, within the promoters of responsive genes. Seminal studies from the Kenney lab revealed that at some promoters, the association of Zta with DNA is dependent on CpG methylation (Bhende et al., 2004, 2005; Dickerson et al., 2009). This key observation led to the recognition of different categories of ZRE, depending on the presence of a CpG-dinucleotide in the sequence. The class I (Karlsson et al., 2008) or simple ZREs (Bergbauer et al., 2010), do not contain a CpG and the binding of Zta is independent of methylation. Class III (Karlsson et al., 2008) or Me-ZREs (Bergbauer et al., 2010) do contain a CpG and the binding of Zta is strictly dependent on methylation. At a minority of ZREs, referred to as class II (Karlsson et al., 2008), DNA methylation has an intermediate impact. Importantly, this classification scheme also applies to ZREs in the host cell genome. For example, *Egr1*, which is activated by Zta (Kim et al., 2007) contains a CpG-ZRE that is methylation dependent (Heather et al., 2009). It is not known whether additional mechanisms are in place to aid Zta activation of DNA-methylated compared to non-methylated promoters.

Zta expression is restricted to two phases of the EBV life cycle; immediately after infection and during the EBV lytic replication cycle. Zta is not expressed during viral latency, indeed enforced expression of Zta promotes cells to initiate the lytic replication cycle. Following physiological stimulation of cells harboring latent EBV, Zta is the first viral lytic replication cycle gene to be expressed and then activates the expression of many viral genes. Zta is expressed initially when the viral genome is heavily methylated and remains expressed when the genome is largely non-methylated. Zta interacts with several hundred sites on the viral genome and at about half of these site binding is dependent on the DNA methylation status (Bergbauer et al., 2010; Flower et al., 2011; Ramasubramanyan et al., 2012a). Many of them occur within important promoters that control the expression of genes essential for the EBV lytic replication cycle (Bergbauer et al., 2010; Flower et al., 2011; Ramasubramanyan et al., 2012a,b). Thus, a sub-set of viral lytic replication cycle promoters is dependent on DNA methylation for activation by Zta (**Figure 1**). This could explain the requirement for genome methylation during the EBV life cycle.

It is puzzling to understand how these methylation-dependent promoters evolved. Why is it advantageous to encode a transcription factor with both methylation-dependent and -independent recognition sites if both classes of ZRE should be equally “visible” to Zta in the methylated state? To understand the driving force behind the differential binding of Zta at ZREs, we need to consider the situation where the EBV genome is non-methylated and the CpG-ZREs become “invisible” (**Figure 1**).

## RELEVANCE OF THE NON-METHYATED EBV GENOME

There are two stages in the life cycle of EBV when the differential recognition of methylation sensitive and insensitive ZREs in promoters could occur; in both the viral genome is non-methylated and Zta is expressed (**Figure 1**).

(i) During the late stage of the EBV lytic replication cycle, large numbers of non-methylated viral genomes and Zta protein accumulate within the nucleus. Whether the demethylation occurs via an active or passive process has not been determined. However, it is clear that Zta interacts with the non-methylated EBV genomes that are present during late lytic cycle (Ramasubramanyan et al., 2012a). Indeed, genome-wide comparisons of Zta binding sites revealed that methylation-independent ZREs are preferentially recognized at this stage (Ramasubramanyan et al., 2012a). This suggests that there could be a switch in Zta-orchestrated gene expression between the early and late stages of lytic replication cycle but this will require further investigation.

(ii) Immediately following infection of cells, the non-methylated EBV genome enters the nucleus, accompanied by a transient burst of Zta expression (Wen et al., 2007; Halder et al., 2009; Kalla et al., 2010). The short-lived nature of this event has thus far precluded a biochemical analysis of Zta binding patterns, but it is clear that only a sub-set of the lytic cycle genes are expressed at this stage and there is no associated generation of infectious virions (Halder et al., 2009; Shannon-Lowe et al., 2009; Kalla et al., 2012). This phase has been termed an abortive lytic cycle or pre-latency step (Woellmer and Hammerschmidt, 2013) and it is postulated that the lack of DNA methylation on the viral genome prevents Zta from activating the full set of lytic replication cycle genes. The advantage to the virus might be that the expression of a limited set of genes provides a boost to the growth or survival of infected cells prior to latency becoming fully established. Indeed, Zta is known to activate the expression of host cytokine genes (Murata and Tsurumi, 2013; Woellmer and Hammerschmidt, 2013) and has a role in the development of lymphomas in a model system (Ma et al., 2011).

## CONCLUSION

The EBV genome provides an exception to the epigenetic paradigm of DNA methylation correlating with a silencing of gene expression. The virus also exploits a unique transcription factor to activate genes embedded in methylated DNA. The ability of Zta to differentially recognize methylated sequence elements together with the biphasic methylation cycle of the viral genome suggest that the selection of these properties was driven by the need to differentially regulate binding to different sub-sets of ZREs. Indeed Zta expression during the pre-latency stage and the lytic cycle results in the expression of different sub-sets of target genes, these are related to the location of methylation-dependent or -independent ZREs in their promoters and the methylation status of the viral genome.

## Conflict of Interest Statement

The author declares that the research was conducted in the absence of any commercial or financial relationships that could be construed as a potential conflict of interest.
